# Liver Transcriptome Profiling Identifies Key Genes Related to Lipid Metabolism in Yili Geese

**DOI:** 10.3390/ani13223473

**Published:** 2023-11-10

**Authors:** Huajiao Dong, Jie Zhang, Yingying Li, Hafiz Ishfaq Ahmad, Tiantian Li, Qianqian Liang, Yan Li, Min Yang, Jilong Han

**Affiliations:** 1 College of Animal Science and Technology, Shihezi University, Shihezi 832061, China; dhj2838132394@163.com (H.D.); lanmao_515@outlook.com (J.Z.); 16627269579@163.com (Y.L.); sweetsweet2022@126.com (T.L.); liangqianqian0224@163.com (Q.L.); liyan_10242023@163.com (Y.L.); 2 Department of Animal Breeding and Genetics, Faculty of Veterinary and Animal Sciences, The Islamia University of Bahawalpur, Bahawalpur 63100, Pakistan; ishfaq.ahmad@iub.edu.pk

**Keywords:** Yili goose, liver, lipid metabolism, glucose metabolism, RNA-seq

## Abstract

**Simple Summary:**

Lipid is an important research topic in geese, and glucose metabolism is inevitably closely related to lipid metabolism. The liver is usually used as the study material in lipid and glucose metabolism. At present, chicken, duck, and goose are the three most widely farmed poultry species around the world, and while there is a wealth of research on lipid and glucose metabolism in the livers of chickens, studies on the liver of domestic geese remain relatively limited. In this study, we studied the liver transcriptome difference between Yili geese with a low body lipid content and their hybrid geese to analyze the differentially expressed genes in the livers using RNA-seq technology, and identified genes related to lipid and glucose metabolism, including *ELOVL6*, *ACOT7*, *ADCY10*, *DGAT1*, *DHCR24*, *HMGCR*, *FDFT1*, *ENO2*, *G6PC3,* and other genes. The results reveal differences in lipid and glucose metabolism in the liver of two types of geese and provide a theoretical basis for the development of methods to modulate lipid metabolism by gene regulations.

**Abstract:**

The Yili goose is the only indigenous goose breed that originates from *Anser anser* in China, known for its adaptability, strong flying ability, and tender meat with a low body lipid content. The liver plays a crucial role in lipid and glucose metabolism, including the intake, secretion, transportation, and storage of fatty acids (FAs). In this study, RNA-sequencing (RNA-seq) technology was performed to analyze the liver differentially expressed genes of Yili geese and their hybrid geese to investigate differences in liver lipid and glucose metabolism. A total of 452 differentially expressed genes (Q-value < 0.05) were identified. Notably, in KEGG enrichment analysis, four pathways (Q-value < 0.05) were enriched to be associated with lipid and glucose metabolism, including the metabolic pathway, PI3K–Akt signaling pathway, glycolysis/gluconeogenesis, and steroid biosynthesis. This study provides insights into potential candidate genes and metabolic pathways that affect the liver lipid metabolism of Yili goose. These findings provide a better understanding of animal liver lipid deposition and metabolism.

## 1. Introduction

Domestic goose is one of the most important economic domesticated poultry breeds in the world, providing humans with products such as meat, fatty liver, eggs, feathers, etc. Goose meat is rich in high-quality protein, unsaturated and essential fatty acids, and has low cholesterol content [1]. Different breeds of domestic geese have significantly different genetic composition, which affects different physiological characteristics. The Yili goose is the only indigenous goose breed that originates from Greylag Goose (*Anser anser*) in China, which is mainly distributed in Yili City and the surrounding areas, Xinjiang, and is known for its adaptability, disease resistance, strong-flying ability, and tender meat with low lipid content [2]. In addition, the Yili goose has a slow growth rate and low abdominal fat rates [3,4]. However, selection on performance does not fully explain why Yili geese have a lower performance than other commercial and native domesticated breeds.

Crossbreeding between a local breed and a high-performance breed is a widespread strategy used to increase growth performance, egg production, etc., in poultry [5]. The utilization of heterosis has long been one of the main objectives of poultry breeders, and in hybridization, a first-generation (F1) hybrid receives half of its genetic material from each of its parents. Typically, F1 hybrids may have heterosis in a wide range of traits, such as environmental adaptation or growth condition. In this study, we try to answer the reason why the Yili goose still retains its unique original growth and performance and low-fat rate. One commercial goose breed, the Sichuan white goose, is famous for its growth performance and high abdominal fat rate [6,7,8], with a significant difference compared to the Yili goose. The distinctions in the climatic environment between Xinjiang and Sichuan result in noticeable contrasts in the genetic background between Yili geese and Sichuan white geese, which may cause additional interference to research activities based in Xinjiang. Consequently, a hybridization program was conducted, using the Yili goose as the male parent and the Sichuan white goose as the female parent. The first-generation hybrid geese were selected as the control group to minimize the divergence in genetic background while preserving the utmost variety among species. The generated hybrid geese still exhibit rapid growth and increased lipid content in the same growth environment as Yili geese, making it a suitable control model for studying the lipid and glucose metabolism of Yili geese. Consequently, genome-wide transcriptome analysis in the liver was used to compare between purebred Yili goose and hybrids. This research aimed to detect DEGs and biological pathways critical for lipid metabolism. Its primary goal was to shed light on the factors that enable Yili geese to maintain their unique original growth and performance characteristics, notably their low fat rate.

The liver regulates animal metabolism, body development, and immune function [9,10]. In poultry, the liver is the primary site for lipid synthesis, secretion, glycogen decomposition, synthesis, and storage [9,11]. The liver in domestic goose and chicken has a strong ability to synthesize fatty acids, with over 90% of fatty acids being synthesized de novo in the liver [9,12]. The liver acts as a central hub where the metabolism of sugars, lipids, and proteins intersect, making it a crucial link in the overall metabolism [13]. To summarize, the livers from the Yili goose and its hybrid geese were chosen with priority to study the molecular mechanism of lipid metabolism and fat deposition. Transcriptome is an efficient way to reflect the gene expression and functional differences of the liver at a certain stage to a certain extent. Lyu et al. [13] used RNA-seq to study differentially expressed genes in pigs with different liver fat contents, providing insights into the gene functions that regulate lipid metabolism. Ge et al. [14] determined differentially expressed genes related to liver transcriptome screening and intramuscular fat formation and lipid deposition in duck meat, and identified marker candidate genes that selectively regulate IMF formation in Chaohu ducks. Gunawan et al. [15] obtained transcriptome profiles of sheep liver using RNA-seq and identified key genes involved in fat synthesis and fat metabolism. However, there is currently limited research on liver lipid and glucose metabolism of geese. Therefore, by comparing the liver transcriptome of Yili geese and their hybrid geese, we can gain a deeper understanding of the genes regulating lipid metabolism in domestic geese and even humans to develop strategies to modulate fat metabolism and explain the reasons of some obesity-related diseases.

## 2. Materials and Methods

### 2.1. Ethical Statement

All animal experiments were authorized by the Biology Ethics Committee of Shihezi University. The ethic committee approval number is: A2020-34.

### 2.2. Animals and Sample Collection

Six five-month-old healthy female geese from the same goose farm with similar body weights were used in this study, including three Yili geese and three hybrid geese. These geese were raised in a shared environment with unrestricted access to food and water. The hybrid geese were produced using Sichuan white geese as the female parent and Yili geese as the male parent. We promptly gathered liver samples from the slaughtered animals, preserved them in liquid nitrogen, and subsequently transferred all tissue samples to a −80 °C refrigerator for storage until they were utilized for total RNA extraction and histological staining.

### 2.3. Histological Staining

Briefly, frozen liver segments of geese were embedded with Tissue-Tek O.C.T compound and then frozen for more than 24 h in a −80 °C refrigerator, producing 5 μm frozen sections [16]. After drying the slices for 2–3 min, they were stained with oil red O for 10 min, then rinsed with running water for 2–3 min, removing excess dye, and then decolorized with 75% ethanol for 10 s until the lipids in the lipid plaques were red, while the rest were colorless [16,17]. Finally, they were stained with hematoxylin for 30–45 s, rinsed with tap water, wiped dry, and sealed with glycerol gelatin [16,18]. A light microscope photographed the liver sections.

### 2.4. RNA Extraction, Library Preparation, and Sequencing

Total RNA from liver tissues samples was isolated using TransZol UP (TransGen Biotech, Beijing, China) and phenol. In brief, RNA library for RNA-seq was prepared as follows: enrich mRNA, reverse transcription, end repair, adaptor ligation to RNA fragments, purify mRNA using oligo (dT)-attached magnetic beads according to kit instructions, and build libraries based on instructions. The integrity of RNA and the library was examined using an Agilent 2100 Bioanalyzer (Agilent Technologies, Santa Clara, CA, USA). Finally, qualified libraries were sequenced using Illumina Novaseq6000 platform at pair-end 150 bp reads (PE150) by Compass Biotechnology Co., Ltd. (Beijing, China). The clean reads were submitted to the Genome Sequence Archive in National Genomics Data Center, China National Center for Bioinformation/Beijing Institute of Genomics, Chinese Academy of Sciences (BioProject: PRJCA019357; GSA: CRA012436).

### 2.5. Quality Control, Comparison, Assembly, and Quantification of Sequencing Data

The raw data from the sequencing were subjected to quality control using the fastp software [19]. The transcriptome sequencing data of each sample after quality control were aligned to the goose reference genome Anser_cygnoides.GooseV1.0.dna.toplevel.fa (https://ftp.ensembl.org/pub/release-110/fasta/anser_cygnoides/dna/, accessed on 3 September 2022) using the HISAT2 (Version: 2.0.4) [20]. After mapping, StringTie software [21] was used to match the unique reads of each sample to the reference genome for transcriptome assembly. Cuffcompare software [22] was used to compare the merged non-redundant transcriptome with the reference genome annotation file to determine the position of each transcript relative to the known gene. Subsequently, FeatureCounts software [23] was used to count the number of reads (counts) that fell within each gene segment in each sample.

### 2.6. Identification of DEGs and Homologous Search of Unknown Genes

DESeq2 (Version 1.40.1) [24] in R software was used to assess the differentially expressed genes (DEGs). The significance was set at |log_2_ (fold change) ≥ 1 and Q-value < 0.05. Homology searches against GenBank databases were performed using the BLAST server (http://www.ncbi.nlm.nih.gov/BLAST/, accessed on 20 March 2023) at the National Center for Biotechnology Information (NCBI) [25]. Homologies that showed identities over 85% and e-values of less than 1 × 10^−5^ with more than 100 nucleotides were considered to be significant [25]. Among the prominent options, the preferred species to choose are birds (chickens, ducks, swans, etc.), and the gene symbol matches the gene description in the NCBI gene database.

### 2.7. Functional Annotation and Pathway Enrichment Analysis of DEGs

Functional annotation of screened differentially expressed genes can help interpret gene function. The g:Profiler (https://biit.cs.ut.ee/gprofiler/gost, accessed on 18 April 2023) [26,27] was used to execute Gene Ontology (GO) enrichment analysis and Kyoto Encyclopedia of Genes and Genomes (KEGG) pathway analysis of the differentially expressed genes (DEGs). After FDR correction, Q-value < 0.05 was considered to be significant. In addition, in order to identify whether differentially expressed genes (DEGs) are involved in the activation or inhibition process, functional annotations were performed separately on up-regulated and down-regulated genes.

### 2.8. Real-Time Quantitative Polymerase Chain Reaction (RT-qPCR) for DEGs Verification

Eight genes were randomly selected for RT-qPCR to verify the accuracy of the transcriptome sequencing data. The total RNA of the liver tissue was extracted using TransZol UP (TransGen Biotech, Beijing, China) and phenol. Then, about 0.1 µg of RNA was reverse-transcribed into cDNA using a SynScript ⅢRT SuperMix for qPCR(+gDNA Remover) Kit (Tsingke, Beijing, China). The synthesized cDNA was used as a template, and reaction mix was configured according to the instructions of ArtiCan^CEO^ SYBR qPCR Mix (Tsingke, Beijing, China). RT-qPCR was then completed using LightCycler 96 (Roche, Basel, Sweden). The glyceraldehyde phosphate dehydrogenase (GAPDH) gene was used as an internal reference genes [28]. Primer-BLAST designed the primers used for quantification in the study on the NCBI website (https://www.ncbi.nlm.nih.gov/tools/primer-blast/, accessed on 3 July 2023). The primer information for RT-qPCR are listed in Table S1. After the reaction was completed, a melting curve analysis was performed. The 2^−∆∆CT^ method was used to analyze the changes in relative gene expression from RT-qPCR experiments [29].

### 2.9. Statistical Analysis

The data were analyzed using GraphPad Prism version 9 and R. The Pearson correlations of DEGs between RT-qPCR and RNA-seq were calculated using the R. The results are expressed as the mean and standard error of the mean (SEM). All significance was declared for *p*-value < 0.05.

## 3. Results

### 3.1. Lipid-Related Phenotypes of Geese

**Figure 1:**
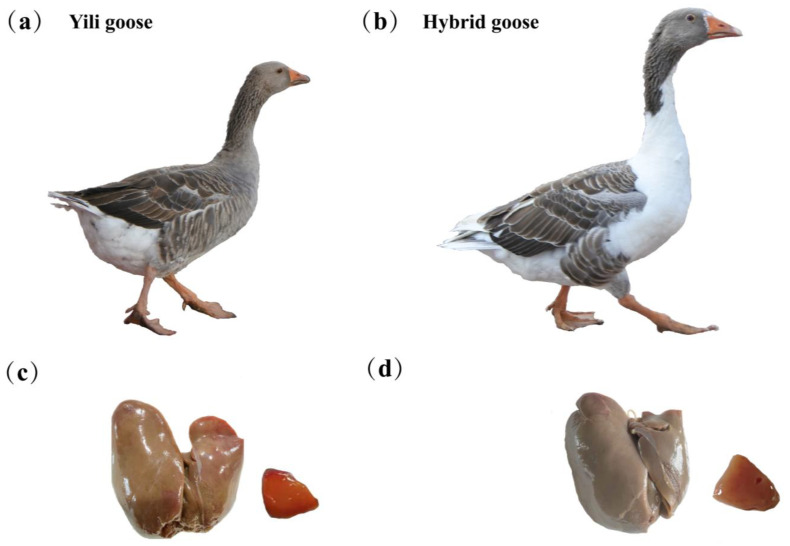
Yili goose and hybrid goose, as well as their hepatic and hepatic segment. (**a**) Yili goose; (**b**) hybrid goose; (**c**) hepatic and hepatic segment of Yili goose; (**d**) hepatic and hepatic segment of hybrid goose.

**Figure 2:**
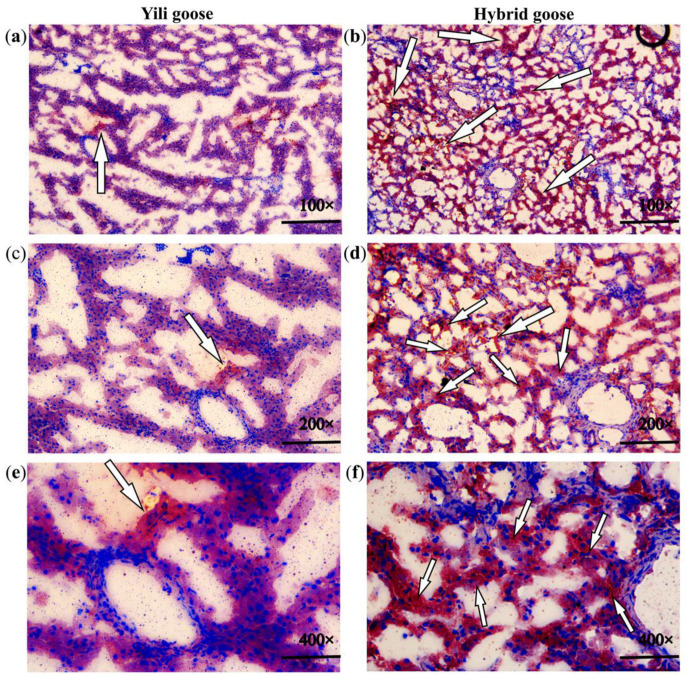
Oil red O staining of liver tissue. By staining with oil red O, the lipid droplets turned orange red to bright red, and the nucleus was stained blue with hematoxylin. (**a**) The enlarged images of Yili goose liver viscera stained with oil red O at 100×; (**b**) The enlarged images of hybrid goose liver viscera stained with oil red O at 100×; (**c**) The enlarged images of Yili goose liver viscera stained with oil red O at 200×; (**d**) The enlarged images of hybrid goose liver viscera stained with oil red O at 200×; (**e**) The enlarged images of Yili goose liver viscera stained with oil red O at 400× magnification; (**f**) The enlarged images of hybrid goose liver viscera stained with oil red O at 400× magnification.

From an external perspective, compared with Yili geese, the livers of the hybrid geese appear to be yellowish in color, with a greasier texture (Figure 1). It is preliminarily determined that the fat content of the liver of hybrid geese is much higher than that of Yili geese. To assess the liver lipid content, we performed oil red O staining on liver segments. As shown in Figure 2, the hybrid geese exhibit more severe lipid accumulation with much more numerous red-stained areas than Yili geese. It is evident that liver lipid content in hybrid geese is higher than that in Yili geese.

### 3.2. Summary of RNA-Seq Data

After quality control of the original reads, an average of 42,275,877 clean reads per sample was generated (ranging from 40,008,006 bp to 43,270,170 bp for Yili geese and 39,803,768 bp to 49,126,426 bp for hybrid geese). The percentage of clean reads of each sample accounting for the original reads is more than 99.22%. The Q20 values range between 94.10% and 97.19%. The Q30 values are between 86.81% and 92.64%. The samples exhibit good quality, with Q20 > 90% and Q30 > 85%. The average number of clean bases is 6.215 G, equivalent to over 6 G. All clean readings of the samples were mapped to a unique reference genome, exceeding 77.75%. Table 1 shows the summary of sequence quality and alignment information from liver transcriptome sequencing of Yili geese and hybrid geese.

### 3.3. Differentially Expressed Genes Analysis

**Figure 3:**
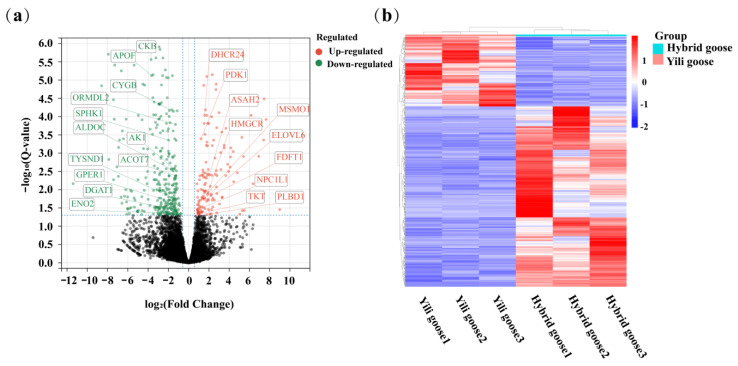
(**a**) Volcano plot of the total expression of genes in both the Yili geese and hybrid geese groups. A total of 18,714 genes were expressed in both the Yili geese and hybrid geese groups, and there were 452 differentially expressed genes (DEGs). The x-axis represents the log2 (fold change) values for gene expression, and the y-axis represents the −log10 significance of the difference in the expression (Padj < 0.05). Red dots indicate 127 up-regulated DEGs, green dots indicate 325 down-regulated DEGs, and gray dots indicate non-differentially expressed genes. (**b**) Liver tissue expression profiles of 452 differentially expressed genes (DEGs) in the Yili geese and hybrid geese groups. Hierarchical clustering analysis of z-scored counts was performed for each DEG between geese in the Yili geese and hybrid geese groups. Color scale represents counts normalized log10 transformed counts. Horizontal bars represent genes. The vertical column represents samples. Red color indicates up-regulated genes, while blue color indicates down-regulated genes.

A total of 452 mRNAs showed differential expression (the average counts of six samples > 5) between Yili geese and their hybrid geese, with 127 up-regulated and 325 down-regulated genes (Table S2). These findings were observed when comparing the gene expression profiles of Yili geese with their hybrid geese (purebred vs. F1 generation), as shown in Figure 3. The expression patterns of differentially expressed genes (DEGs) in each sample were clustered based on their expression ratios’ log2 (fold change) values. This analysis demonstrates the consistent and repeatable expression patterns within the two groups of samples, as depicted in Figure 3.

### 3.4. GO Annotation and Enrichment Analysis of Differentially Expressed Genes

**Figure 4:**
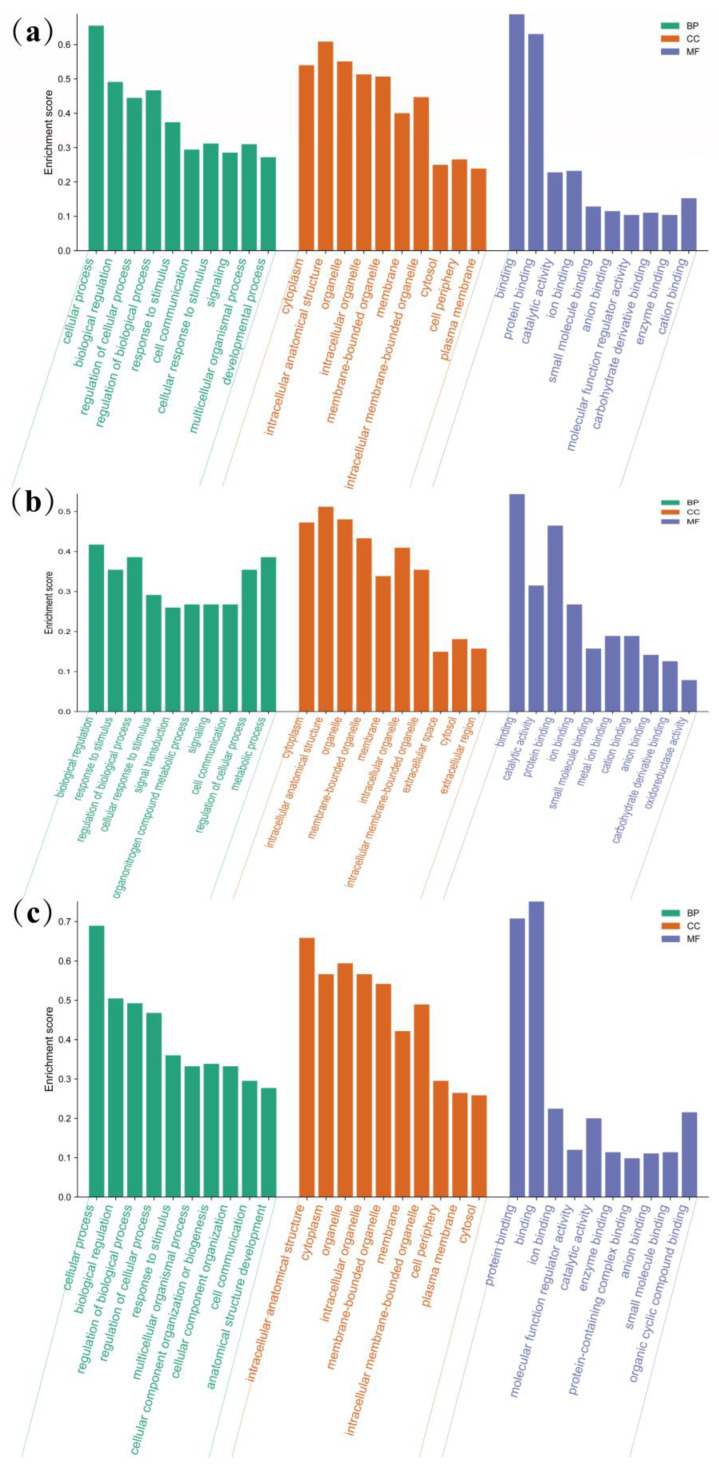
GO enrichment analyses of the DEGs in livers of Yili geese and its hybrid geese. The GO terms include biological processes (BPs), cellular components (CCs), and molecular functions (MFs); (**a**) all DEGs; (**b**) up-regulated DEGs; (**c**) down-regulated DEGs.

To further investigate the functional roles of the 452 differentially expressed genes (DEGs), a GO term enrichment analysis was conducted to identify significantly over-represented categories. In order to further identify the activation or inhibition function of genes, up-regulated and down-regulated genes were identified separately by GO term enrichment analysis. A total of 350 DEGs were found to be enriched in GO terms. GO annotation includes three classifications: biological processes (BPs), cellular components (CCs), and molecular functions (MFs), GO terms for all genes, up-regulated genes, and down-regulated genes are included in Figure 4 and Table S3.

**Figure 5:**
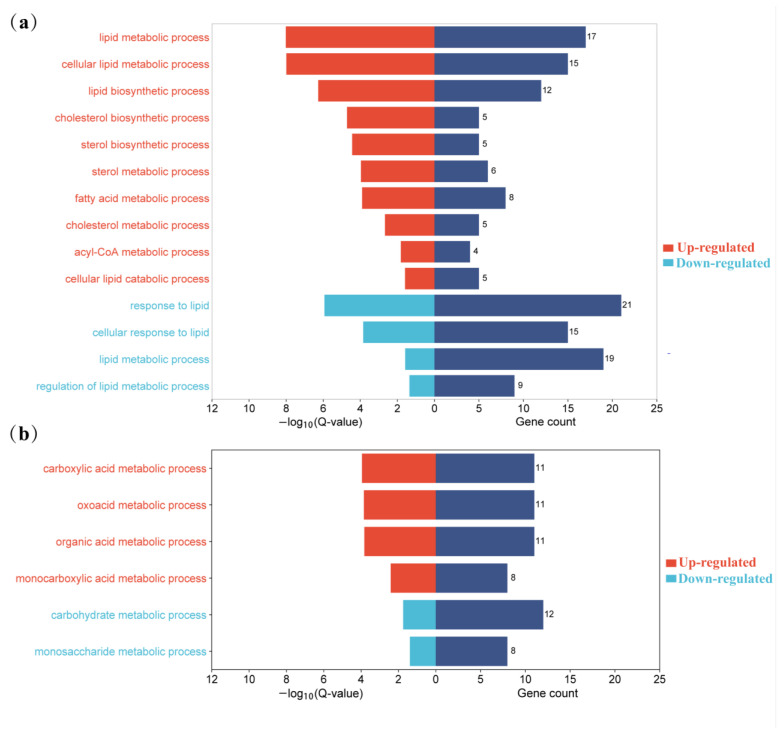
The significantly enriched GO terms associated with lipid and glucose metabolism in up-regulated and down-regulated genes. (**a**) GO terms associated with lipid metabolism; (**b**) GO terms associated with glucose metabolism.

Regarding BPs, a total of 318 DEGs were enriched to 478 terms (Q-value < 0.05). These included “metabolic process”, “regulation of cellular process”, and “organic substance metabolic process”. Regarding CCs, 335 DEGs were enriched, and 103 terms exhibited significant enrichment (Q-value < 0.05). Examples of significantly enriched terms in CCs were “endoplasmic reticulum”, “mitochondrion”, and “transporter complex”. For MFs, 331 DEGs were enriched, and 81 terms showed significant enrichment (Q-value < 0.05). Some of the significantly enriched terms in MFs included “protein binding”, “catalytic activity”, and “oxidoreductase activity”. The terms related to lipid metabolism and glucose metabolism have been significantly enriched, and the results are presented in Table 2 and Table 3. Figure 5 shows the significantly enriched GO terms associated with lipid and glucose metabolism in up-regulated and down-regulated genes. Up-regulated genes were enriched in the biosynthesis and metabolism of cholesterol and sterol, the metabolism of fatty acids and acyl-CoA, the cellular lipid catabolic process, and organic acid metabolic process. Down-regulated genes were related to lipid metabolism, response to lipid, regulation of lipid metabolism, the monosaccharide metabolic process, and carbohydrate metabolic process. The genes of significantly enriched GO terms associated with lipid and glucose metabolism in up-regulated and down-regulated genes are shown in Table S4.

### 3.5. KEGG Pathway Analysis of DEG

**Figure 6:**
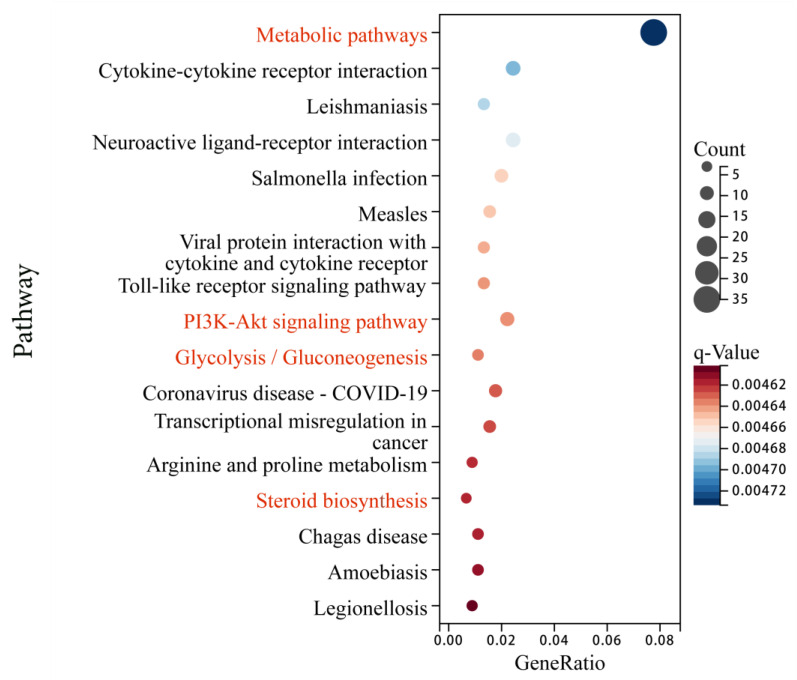
Significantly enriched KEGG pathways of DEGs (Q-value < 0.05). The abscissa represents the rich ratio, and the ordinate represents the pathway name.

To identify the pathways those DEGs involved, we integrated the 452 DEGs into the KEGG pathway database, and a total of 17 pathways (Q-value < 0.05) were significantly enriched (Figure 6, Table S3). Among them, four pathways are involved in lipid and glucose metabolism, including metabolic pathways, the PI3K–Akt signaling pathway, glycolysis/gluconeogenesis, and steroid biosynthesis. Up-regulated genes were enriched into metabolic pathways and steroid biosynthesis, while down-regulated genes were related to metabolic pathways, the PI3K–Akt signaling pathway, and glycolysis/gluconeogenesis.

### 3.6. Gene Screening and Protein Interaction Analysis

**Figure 7:**
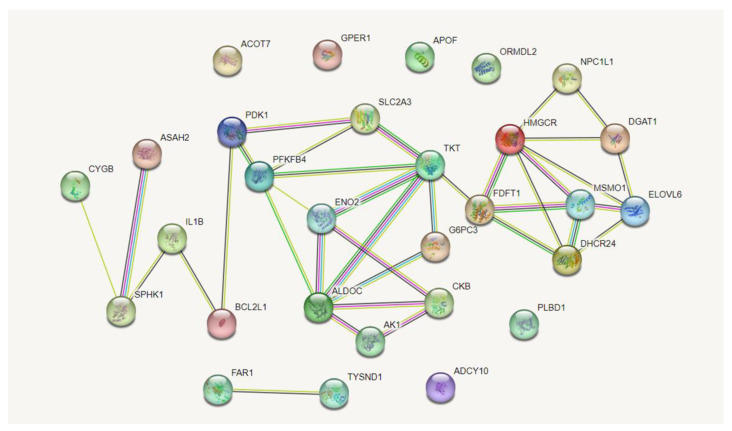
PPI network of DEGs. In the network diagram, nodes represent various proteins, and node labels are the names of these proteins. The patterns in the nodes represent the three-dimensional structure of the protein. Suppose there is an interaction between two proteins. In that case, they are connected by a line, and the color of the line reflects the type of interaction, including experimentally verified or predicted, as well as direct physical interactions, co-expression, gene fusion, and other relationships. The thicker the line, the stronger the interaction between the two proteins.

Through GO and KEGG functional enrichment analysis, KEGG and GO entries related to lipid and glucose metabolism were identified in the liver of Yili geese and their hybrid geese (Table 3 and Table S4, Figure 5 and Figure 6). The genes contained in these entries were queried in Pathcards [30] and 29 DEGs related to lipid and glucose metabolism were selected (Table S5). Among these genes, 12 genes (*DHCR24*, *ELOVL6*, *ASAH2*, *MSMO1*, *FDFT1*, *TKT*, *HMGCR*, *ADCY10*, *PDK1*, *PLBD1*, *NPC1L1*, *FAR1*) were up-regulated, and 17 genes (*AK1*, *ALDOC*, *CKB*, *ENO2*, *DGAT1*, *G6PC3*, *BCL2L1*, *GPER1*, *CYGB*, *TYSND1*, *IL1B*, *ORMDL2*, *APOF*, *SLC2A3*, *PFKFB4*, *SPHK1*, *ACOT7*) were down-regulated. Then, we used STRING [31] to perform protein–protein interaction analysis on these 29 genes and obtained a PPI network diagram (Figure 7). From protein–protein interaction analysis, it can be seen that most genes have protein–protein interactions. Although the interactions between ADCY10, APOF, ACOT7, GPER1, and ORMDL2 were not found in the protein–protein interaction database, they may also play an important role in glucose and lipid metabolism.

### 3.7. RNA-Seq Data Validation by RT-qPCR

**Figure 8:**
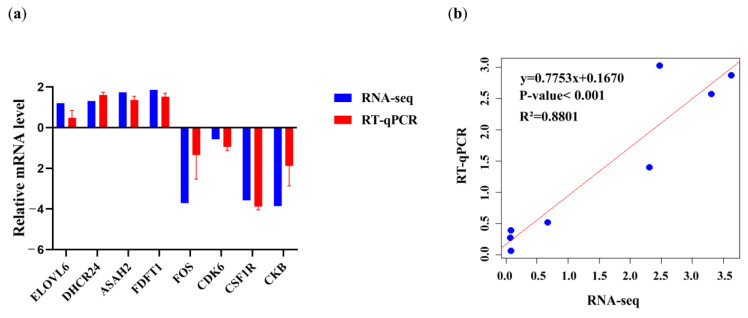
(**a**) Validation of RNA-seq results via RT-qPCR; (**b**) Pearson correlation analysis and construction of univariate linear regression.

In order to verify the authenticity of the RNA-seq data of Yili geese and its hybrid geese, RT-qPCR was performed on eight DEGs. As shown in Figure 8, it is found that the expression patterns of these eight genes are consistent with those in RNA-seq results: *DHCR24*, *ELOVL6*, *ASAH2*, and *FDFT1* are up-regulated in the livers of Yili geese, and *FOS*, *CDK6*, *CSF1R*, and *CKB* are down-regulated in the livers of Yili geese (Pearson’s (R^2^ = 0.8801).

## 4. Discussion

The growth and performance of poultry are mostly determined by genetics and environment. Research has shown that migratory birds have strong fat deposition ability in their liver due to migration needs, and domestic geese, as descendants of migratory birds, have good liver fat deposition ability [11,32]. Many domestic goose breeds also have high fatty deposition ability in their liver [33]. The liver is an important organ for animal nutrition, metabolism, and transport. The liver’s metabolic regulation is very complex, influenced by factors such as nutrition, environment, and genetics [34]. The Yili goose has a low abdominal fat rate [3,4], making it a suitable model for studying the mechanism of lipid and glucose metabolism. Considering the importance of F1 heterosis in breeding, in our present study, liver transcriptome between Yili geese and their hybrid geese was performed. Our results suggest that the identified DEGs like *ELOVL6*, *ADCY10*, *DGAT1*, etc., and the enrichment pathways, like the PI3K–Akt signaling pathway and glycolysis/gluconeogenesis pathways play an important role in underlying lipid traits in geese.

**Figure 9:**
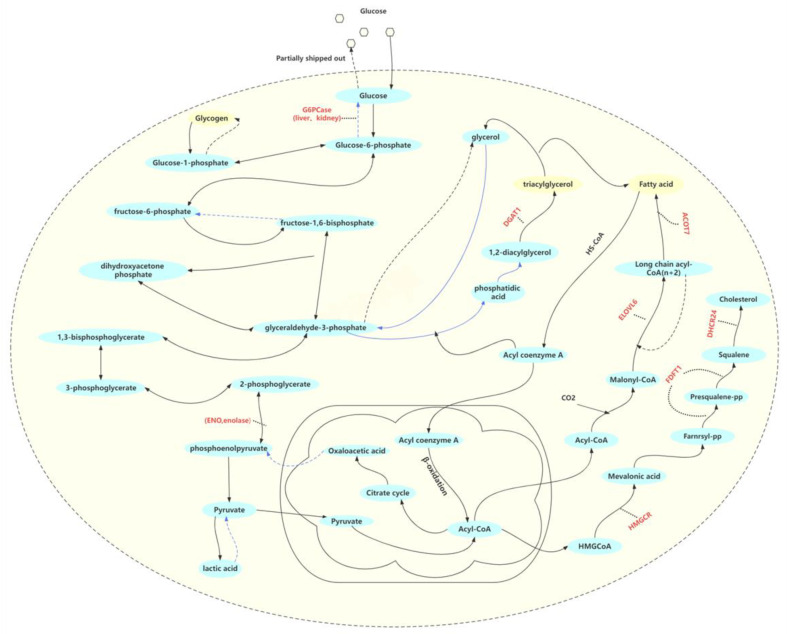
The relationship between lipid metabolism and glucose metabolism. The genes with a red color are differentially expressed genes involved in the metabolic process, while yellow is an important energy substance in the liver. Different linear connections between the two substances represent the different reaction conditions required for their mutual transformation (such as different enzymes). This figure refers to the KEGG pathway (map00010, map00561, map00062, N01635) and the references in this article [38,48,50].

In our study, 452 genes were found to be differentially expressed (127 were up-regulated and 325 were down-regulated) between Yili geese and the hybrid geese. There were four KEGG pathways involved in lipid metabolism and glucose metabolism, including metabolic pathways, PI3K–Akt signaling pathway, glycolysis/gluconeogenesis, and steroid biosynthesis. Up-regulated genes were enriched into metabolic pathways and steroid biosynthesis, while down-regulated genes were related to metabolic pathways, the PI3K–Akt signaling pathway, and glycolysis/gluconeogenesis. In GO terms, up-regulated genes were enriched in the biosynthesis and metabolism of cholesterol and sterol, metabolism of fatty acids, cellular lipid catabolism, while down-regulated genes were related to lipid metabolism, response to lipid, regulation of lipid metabolism, and carbohydrate metabolism processes (Figure 9). Protein–protein interaction analysis shows that there is a strong interaction relationship between genes such as ELOVL6, FDFT1, HMGCR, DHCR24, and DGAT1. The disruption of lipid metabolism balance in the liver is the reason for accumulating large amounts of triglycerides. Currently, it is believed that the increased synthesis of triglycerides, fatty acids β-oxidation reduction, reduced synthesis, and the secretion of extremely low-density lipoprotein can all lead to the accumulation of triglycerides in the form of lipid droplets [35,36]. Based on the enrichment analysis of DEGs genes and the results of protein–protein interaction analysis, it suggests that vigorous cholesterol synthesis metabolism, efficient lipid transport capacity, triglyceride, and carbohydrate utilization abilities may be crucial for maintaining low abdominal fat and low hepatic fat in Yili geese, ultimately resulting in the difference in liver lipid content between Yili geese and hybrid geese.

Maintaining normal cholesterol metabolism is closely related to the secretion of liver lipids. Hydroxy-3-methylglutaryl-CoA reductase (HMGCR) is the rate-limiting enzyme for cholesterol synthesis and is involved in the synthesis of mevalonic acid [37]. Farnesyl diphosphate farnesyl transferase1 (FDFT1) is crucial in converting farnesyl diphosphate to pre-squalene diphosphate and then to squalene. The last step in the cholesterol synthesis pathway, 24 dehydrocholesterol reductase (DHCR24), catalyzes the conversion of lanosterol and other intermediates into cholesterol [38]. Therefore, the up-regulated genes of *FDFT1*, *HMGCR*, and *DHCR24* are involved in the cholesterol synthesis and metabolism in Yili geese, which is of great significance for maintaining cholesterol metabolism balance. In addition, up-regulated genes were also enriched in fatty acid metabolism, the acyl-CoA metabolic process, and organic acid metabolism processes, reflecting the extremely strong metabolic ability of Yili geese. *ELOVL6*, as an important gene for lipid synthesis [39], is also highly expressed in Yili geese. During the migration process of migratory birds, fatty acids are the main source of energy. Before migration begins, the content of fatty acids synthesized by the liver will greatly increase [11,32,40]. Therefore, lipid synthesis is crucial for migratory birds, like wild geese. However, based on our current results, we still cannot fully understand these complex regulatory mechanisms or why Yili goose have such low fatty content both in liver and other body tissues compared with other domestic geese. Surprisingly, the soluble adenylate cyclase (sAC) encoded by *ADCY10* may also play a role in fatty acid metabolism, increasing fatty acid metabolism β-oxidation [41]. β-Oxidation and lipoprotein secretion can reduce liver lipid content [42]. In our study, *ADCY10* was also highly expressed in Yili geese, while it was almost not expressed in hybrid geese. As mentioned above, *ADCY10* may promote fatty acid oxidation and lipid utilization, collaborate with *ELOVL6* to provide energy for their behavers like flight in Yili geese, and play an important role in maintaining cholesterol metabolism balance in Yili geese. Many genes related to calcium ion transport are also up-regulated in Yili geese, while *ADCY10* is highly expressed in Yili geese and down-expressed in hybrid geese. This evidence suggests that *ADCY10* could be one of the key regulating switches for the energy metabolism in Yili goose.

**Figure 10:**
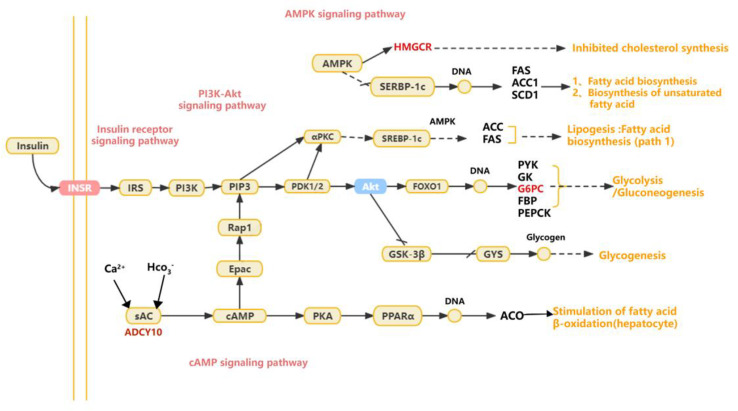
The predicted signal pathways affect liver lipid and glucose metabolism. The genes with a red color are differentially expressed genes involved in the signaling pathway, while the orange text represents the biological processes ultimately affected by the signaling pathway. The solid line represents direct influence, while the dashed line represents the omission of many intermediate processes. This figure refers to the KEGG pathway (map04151, map04910, map04024, map04152) and the references in this article [34,37,40,43,44,47].

The down-regulated genes were enriched in the PI3K–Akt and glycolysis/gluconeogenesis pathways, etc. (Figure 10). Research has shown that the PI3K–Akt signaling pathway plays a role in maintaining lipid metabolism and glucose metabolism homeostasis [34,43], which is consistent with our results in KEGG enrichment. PI3K is a key mediator for insulin to function [44,45], while insulin is a hormone closely related to lipid and glucose metabolism, which can promote the synthesis of glycogen, lipids, and fatty acids as well as reduce the secretion of low-density lipoprotein and inhibit the oxidation of fatty acids [46,47]. From this perspective, the normal operation of the PI3K–Akt signaling pathway is also crucial for maintaining lipid metabolism balance in Yili geese. This means that the genes included in the down-regulation pathway are consistent with those in down-regulated GO term of glucose metabolism, such as *G6PC3*, *ENO2*, and *ALDOC*, and may play a positive role in promoting glycolysis and lipid deposition. Akt can affects enzyme activity (G6PC3) through FoxO1 and GSK3 β phosphorylation, thereby inhibiting gluconeogenesis, reducing glycogen synthesis, and accelerating glycolysis [44,45]. *G6PC3* and *ENO2* have higher expression levels in hybrid geese; perhaps they play a role in promoting glycolysis, inhibiting glucose metabolism, and then affecting liver fat metabolism (Figure 9). Go enrichment analysis of down-regulated genes shows that *ACOT7* and *DGAT1* are associated with lipid metabolism regulation, and the increase in triglyceride synthesis may be related to the *DGAT1* gene. Under the action of a series of enzymes, glucose can be converted into dihydroxyacetone phosphate and glycerol, ultimately producing triglycerides. In the diacylglycerol pathway, 1,2-diacylglycerol is converted into triglycerides under the action of diacylglycerol O-acyltransferase1 (DGAT1) [48,49]. Related studies show that over-expression of *DGAT1* ultimately leads to an increase in triglycerides in mouse adipose tissue and skeletal muscle [50]. Over-expression of the *DGAT1* gene can significantly promote triglyceride formation and lipid deposition in goat precursor adipocytes [49]. The expression level of *DGAT1* is higher in hybrid geese than in Yili geese, which is consistent with the phenotype of increased lipid accumulation in the liver of hybrid geese. In other animals, *DGAT1* positively affects lipid accumulation, indicating that it is one of the candidate genes with high lipid content in the liver of hybrid geese. As mentioned in an earlier text, there is a strong interaction relationship between DGAT1, ELOVL6, FDFT1, HMGCR, and DHCR24, and the functions of *DGAT1* vs. *ELOVL6*, *FDFT1*, *HMGCR*, and *DHCR24* appear to be antagonistic in some cases. These results suggest that the selected candidate genes screened are not individually driven, but interact with each other during the metabolic process, attempting to achieve a dynamic balance of fat synthesis and utilization to maintain the lipid homeostasis.

## 5. Conclusions

In this study, we analyzed the DEGs in geese liver between Yili and their hybrids. Our current results show that the PI3K–Akt signaling and glycolysis/gluconeogenesis pathways play an important role in lipid metabolism in geese, and the DEGs including *ELOVL6*, *ACOT7*, *ADCY10*, *DGAT1*, *DHCR24*, *HMGCR*, *FDFT1*, *ENO2*, and *G6PC3* display a crucial regulatory role in lipid and glucose metabolism in geese livers. Therefore, this study provides insights into potential candidate genes and metabolic pathways that affect the liver lipid metabolism of the Yili goose. These findings provide a better understanding of animal liver lipid deposition and metabolism.

## Figures and Tables

**Table 1:** The basic statistics for RNA-seq reads were generated from the livers of Yili goose and its hybrid goose.

Sample	Read Numbers (bp)	Clean Reads (bp)	Clean Ratio (%)	Clean Base	GC%	Q20	Q30	Mapped Ratio(%)
Yili goose1	40,245,484	40,008,006	99.41%	6.00 G	47.40	97.19%	92.64%	87.65%
Yili goose2	40,650,512	40,440,622	99.48%	6.07 G	47.29	96.98%	92.11%	87.74%
Yili goose3	43,524,680	43,270,170	99.42%	6.49 G	47.10	96.81%	91.92%	77.75%
Hybrid goose1	41,299,504	41,006,270	99.29%	6.15 G	46.91	94.10%	86.81%	80.06%
Hybrid goose2	40,032,290	39,803,768	99.43%	5.97 G	48.10	97.18%	92.50%	87.52%
Hybrid goose3	49,512,794	49,126,426	99.22%	7.37 G	47.84	96.94%	92.12%	82.73%

**Table 2:** The significantly enriched GO terms associated with lipid metabolism.

Term ID	Description	Q-Value	Gene Number
GO:0006629	Lipid metabolic process	0.010901014	38
GO:0044255	Cellular lipid metabolic process	0.011382412	30
GO:0008610	Lipid biosynthetic process	0.014621669	21
GO:0006631	Fatty acid metabolic process	0.0189958	14
GO:0016042	Lipid catabolic process	0.027932536	11
GO:0035336	Long-chain fatty-acyl-CoA metabolic process	0.032135698	4
GO:0006695	Cholesterol biosynthetic process	0.032585568	4
GO:0006637	Acyl-CoA metabolic process	0.03457691	6
GO:0016126	Sterol biosynthetic process	0.037132117	5
GO:0019216	Regulation of lipid metabolic process	0.037300739	10
GO:0016125	Sterol metabolic process	0.038013044	7

**Table 3:** The significantly enriched GO terms associated with glucose metabolism.

Term ID	Description	Q-Value	Gene Number
GO:0019752	Carboxylic acid metabolic process	0.015848476	23
GO:0043436	Oxoacid metabolic process	0.016400085	23
GO:0006082	Organic acid metabolic process	0.016554431	23
GO:0032787	Monocarboxylic acid metabolic process	0.019241064	17
GO:0005975	Carbohydrate metabolic process	0.02169968	16
GO:0005996	Monosaccharide metabolic process	0.024555726	10
GO:0019318	Hexose metabolic process	0.029380642	9
GO:0006006	Glucose metabolic process	0.032569981	8

## Data Availability

The clean sequence data reported in this paper have been deposited in the Genome Sequence Archive in National Genomics Data Center, China National Center for Bioinformation/Beijing Institute of Genomics, Chinese Academy of Sciences (BioProject: PRJCA019357; GSA: CRA012436) that are publicly accessible at https://ngdc.cncb.ac.cn/gsa (accessed on 28 August 2023).

## Supplementary Materials

The following supporting information can be downloaded at: https://www.mdpi.com/article/10.3390/ani13223473/s1, Table S1: Primers for RT-qPCR; Table S2: The details of differentially expressed genes between Yili geese and hybrid geese; Table S3: Significantly enriched GO terms of DEGs; Table S4: The genes of significantly enriched GO terms associated with lipid and glucose metabolism in up-regulated and down-regulated genes; Table S5: The fat and glucose metabolism related DEGs in the livers of Yili geese vs. hybrid geese.
